# Ultraviolet disinfection (UV-D) robots: bridging the gaps in dentistry

**DOI:** 10.3389/froh.2023.1270959

**Published:** 2023-11-01

**Authors:** Visha Shailesh Pandya, Mohamed S.M. Morsy, Ali Abdel-Halim Abdel-Azim Hassan, Hamed A. Alshawkani, Abdulelah Sameer Sindi, Khurshid A. Mattoo, Vini Mehta, Ankita Mathur, Aida Meto

**Affiliations:** ^1^ Department of Public Health Dentistry, Vaidik Dental College & Research Centre, Dadra and Nagar Haveli and Daman and Diu, India; ^2^ Department of Prosthetic Dental Sciences, College of Dentistry, Jazan University, Jazan, Saudi Arabia; ^3^ Department of Maxillofacial Surgery and Diagnostic Sciences, College of Dentistry, Jazan University, Jazan, Saudi Arabia; ^4^ Department of Restorative Dental Science, College of Dentistry, Jazan University, Jazan, Saudi Arabia; ^5^ Department of Restorative Dental Sciences, College of Dentistry, King Khalid University, Abha, Saudi Arabia; ^6^ Department of Dental Research Cell, Dr. D.Y. Patil Dental College and Hospital, Dr. D.Y. Patil Vidyapeeth, Pune, India; ^7^ Department of Dentistry, Faculty of Dental Sciences, University of Aldent, Tirana, Albania; ^8^ Clinical Microbiology, School of Dentistry, University of Modena and Reggio Emilia, Modena, Italy

**Keywords:** artificial intelligence, COVID-19, disinfection robot, dentistry, robot sterilization, ultraviolet disinfection

## Abstract

Maintaining a microbe-free environment in healthcare facilities has become increasingly crucial for minimizing virus transmission, especially in the wake of recent epidemics like COVID-19. To meet the urgent need for ongoing sterilization, autonomous ultraviolet disinfection (UV-D) robots have emerged as vital tools. These robots are gaining popularity due to their automated nature, cost advantages, and ability to instantly disinfect rooms and workspaces without relying on human labor. Integrating disinfection robots into medical facilities reduces infection risk, lowers conventional cleaning costs, and instills greater confidence in patient safety. However, UV-D robots should complement rather than replace routine manual cleaning. To optimize the functionality of UV-D robots in medical settings, additional hospital and device design modifications are necessary to address visibility challenges. Achieving seamless integration requires more technical advancements and clinical investigations across various institutions. This mini-review presents an overview of advanced applications that demand disinfection, highlighting their limitations and challenges. Despite their potential, little comprehensive research has been conducted on the sterilizing impact of disinfection robots in the dental industry. By serving as a starting point for future research, this review aims to bridge the gaps in knowledge and identify unresolved issues. Our objective is to provide an extensive guide to UV-D robots, encompassing design requirements, technological breakthroughs, and in-depth use in healthcare and dentistry facilities. Understanding the capabilities and limitations of UV-D robots will aid in harnessing their potential to revolutionize infection control practices in the medical and dental fields.

## 1. Introduction

The healthcare and its associated sectors are one of the fastest-growing industries globally. In a health care facility, microorganisms persist on an inanimate surface for a longer period causing transmission of infectious diseases (bacterial, viral and fungal) through direct or indirect contact (1, 2). Novel techniques are essential due to increase in the transmission and fatality rate of viral disease, as one seen during a COVID-19 pandemic that was known to last for 28 days under controlled laboratory environments (3, 4). This calls for making the eradication of all microorganisms on seemingly non infected areas a crucial component of disinfection. Disinfection is a process that halts the dissemination of all infectious agents by inactivating them and preventing their transmission (5). Several hospital settings like wards and theatres need to be cleansed repeatedly in a single day, from donning the right attire to disinfecting, which consumes time using traditional ways. Employees perpetually face a hazard of developing an infection under these subjective ways. According to hospital data, even with stricter standards (6) and more effective cleaning processes, fatal infections are on the rise (7). These numbers indicate that the existing strategy is insufficient to shield susceptible individuals from serious, perhaps fatal infections like SARS-COV-2 (8). The Covid 19 pandemic stretched the limits and endurance of healthcare facilities and workers who managed to somehow cope with the challenges. Perhaps the frequent occurrences of epidemics during the present millennium which has seen more than 70 epidemics (9), played a key role in combatting the covid 19 pandemic. The appeal of using robotic disinfection is gaining traction especially among Hospital administrations, because of automation, economical (decreased labour), increased efficacy (wide spectrum of pathogen), less hazardous residuals and relatively simple procedure in a medical setting (10). According to a number of studies, disinfection methods that use UVD irradiation are superior to those that do not, lowering microbial load in the environment and possibly lowering risk of contracting a healthcare-associated infection (HAI) (11–14).

A mobile UV-D robot that can kill microorganisms was created by Guettari et al. (11). Dancer and King (12) evaluated the effectiveness of UV light-based automatic decontaminating systems. Critical evaluations on UV disinfection were presented by Abajo et al. (15) and Raeiszadeh et al. (16) who also presented a wide array of UV decontamination techniques as well as the effectiveness and security of these UV devices. While Martins et al. (17) studied the effectiveness of various disinfection techniques for COVID-19 in diverse circumstances, Chiappa et al. (18) published a narrative review that illustrated the efficacy of a range of UV disinfecting systems against various coronavirus strains. Various studies that discuss applications of UV-D robots (19–21) to address specific issues encountered during COVID-19 testing, cleaning, and disinfection have also been published.

Nonetheless, the bulk of previously published reviews primarily centered around traditional ultraviolet germicidal irradiation (UVGI) systems, with none addressing the autonomous capabilities of UV-D robots, particularly in the context of medical and dental applications. Although, a new era of robot aid based on artificial intelligence is emerging in dentistry, these robots are still not entirely utilised in dental investigations. Conversely, numerous studies have focused on specific robotic systems within distinct disciplines. For instance, research has delved into the role of robots in tooth preparation within prosthodontics (22), as well as the utilization of arch-wire bending robots in orthodontics (23, 24). Furthermore, significant advancements have been made in applying robotic guidance to dental implant placement in oral and maxillofacial surgery (25–27). The progress in procedures like craniomaxillofacial osteotomy has been notably swift and comprehensive, likely attributed to the rapid development of surgical robotic technologies. In order to thoroughly examine and assess the present situation of practical usage of UV-D robot in dentistry, Yajie Li et al. conducted a scoping assessment of 113 studies in 2021. They came to the conclusion that there are still restrictions and inequalities between robotics research and its use in dentistry (28). While UV-D robots have been extensively studied and utilized in the realms of prosthodontics, orthodontics, and oral surgery (22–27), there remains a notable gap in the literature with regard to their comprehensive role in the disinfection process. To the best of our knowledge, numerous research has been published utilizing disinfectant robot in the recent literature (29–31) and the authors feel that not only it is time to review the findings of these studies but also to review the detailed application of disinfectant robot in dentistry. As a result, the present review sought in determining the current state of robotic dental usage, highlight shortcomings, offer perspectives on their adoption and advancement in the future.

The aim of this review is to emphasize the effective usage of UV-D in dentistry, with specific objectives to comprehensively understand the advancements in technology, design requirements, and applications in healthcare and dental facilities. By accomplishing these objectives, this review seeks to promote further scientific investigations in this emerging and innovative field.

## 2. Description of the instruments and technology

### 2.1. UV-D robots

Robotics, is defined as the study of reprogrammable, multifaceted, versatile, and extensible systems that dynamically connect sensing to action (28). UV disinfection robots work on the principle of UVGI using ultraviolet (UV-C) light which has a wavelength of 254 nm and provides antimicrobial, antifungal and antiviral properties.

### 2.2. UV-D robots in disinfection

**Figure 1:**
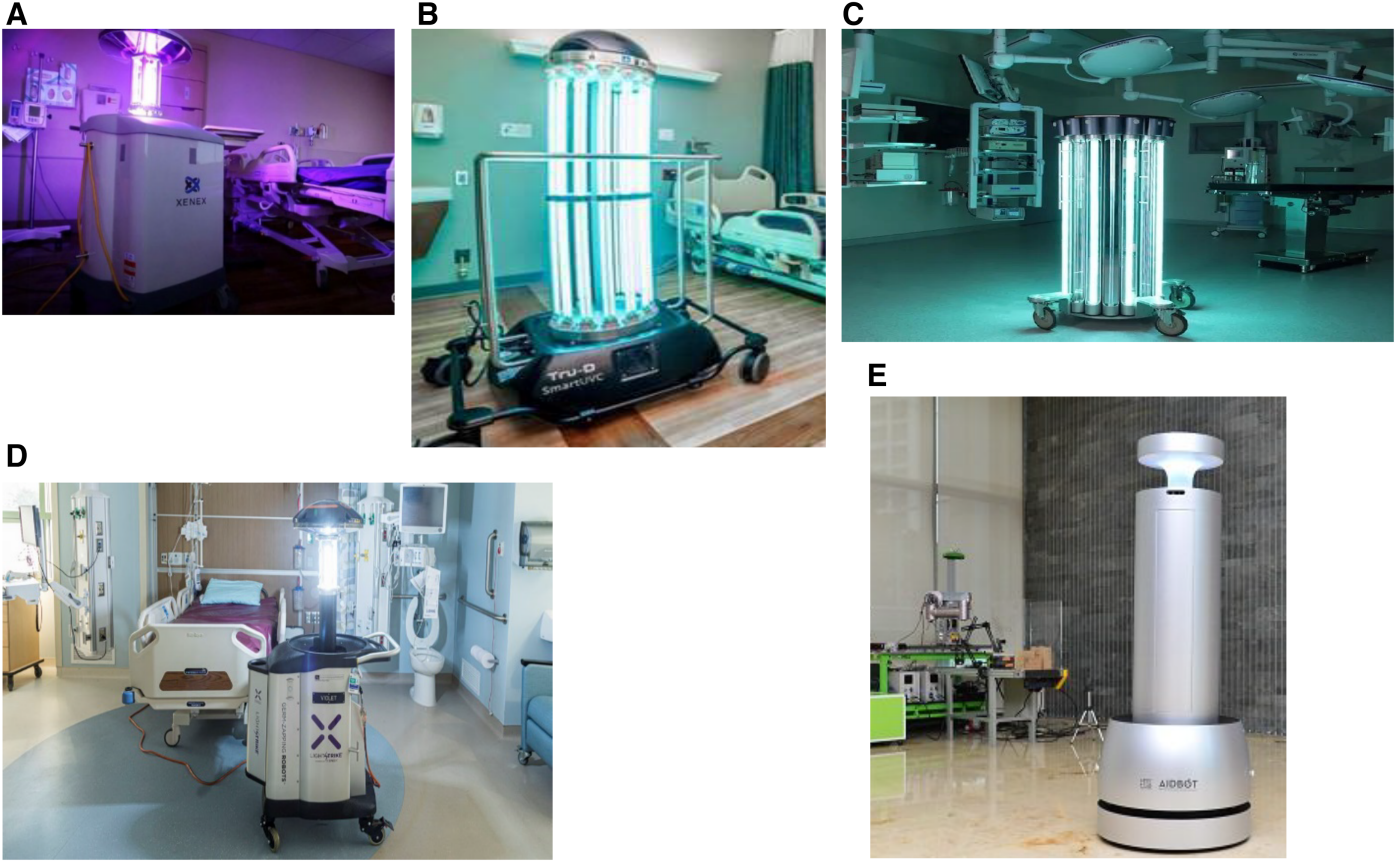
Examples of different ultraviolet disinfection (UV-D) robots. (**A**) Xenex LightStrike-Germ-Zapping Robot. (**B**) Tru-D Smart UVC robot. (**C**) Helios UV-C Disinfection Robot- Surfacide. (**D**) VIOLET. (**E**) Artificial Intelligence Disinfection ROBOT (AIDBOT).

UV-D Robots are powered by batteries and can kill up to 99.99% of germs. These gadgets are mobile base, have a number of Low-pressure Mercury (LPM) lamp, pulsed xenon lamps, and sensors for motion and are used to supplement hand cleaning (11, 32). This robot has various functions, including mapping the area, uses passive infrared sensors to sense its environment, camera with three-dimensional imaging to detect obstructions, choice of manual or automatic operation, and an exceptionally high degree of disinfection. They employ the environment’s map to provide a high-powered UV dose (33), and rely on simultaneous localization and mapping (SLAM) (34), to generate a map of working environment. They can disinfect with UV-C light of 254 nm wavelength, and it includes 8 UV-C lamps disinfecting a 360° coverage area (35). A feature of these gadgets is its sensor that keeps track of environmental factors like temperature and humidity. Additionally, sensors that detect movement are employed to instantly shut UV lamps in the event that any humans are found. Traditional UV-D equipment is often either left in one spot in room for the duration of the disinfection cycle or is manually moved by the designated operator to various locations (5). Various studies have reported that disinfection can benefit from the incorporation of robotics in the management of infectious diseases (21, 36). Table 1 and Figure 1 presents a comparison of UV robots, on the basis of design specifications.

**Table 1:** Comparison of UV robots based on design specifications.

Features	XENEX-Light Strike [European Institute]	TRU-D [European Institute]	HELIOS [The HELIOS]	VIOLET [Violet, Mc Ginn]	AIDBOT [AIDBOT, Gebhart]
Founder	Mark Tuck Stibich	Memphis Tenn	Gunner Lyslo	Conor McGinn	KangGeon Kim
Country origin	USA	San Diego	USA	Ireland	Korea
Year	2014	2020	2020	2020	2021
Compactness for use in confined locations	Absent	Absent	Present	Present	Present
Reduction in majority of microorganisms	*Candida auris*, *C. diff*., VRE and CRE	*C. diff.*, MRSA, VRE, Ebola and Acinetobacter	*C. diff*., MRSA, VRE, Ebola and Acinetobacter	*C. diff.*, MRSA, VRE, Ebola and Acinetobacter, SARS-COV-2	*C. diff.*, MRSA, VRE, Ebola and Acinetobacter, SARS-COV-2
Autonomous Navigation	Absent (uses a separate controller)	Absent	Present (uses scrub feature)	Present	Present (uses a mobile controller)
Feasibility of using a cleaning crew and a robot in the same area.	Absent	Absent	Present	Present	Present

USA, United States of America; MRSA, methicillin-resistant *Staphylococcus aureus*; VRE, vancomycin-resistant *Enterococcus*; CRE, carbapenem-resistant *Enterobacteriaceae*; *C. diff.*, *Clostridioides difficile*.

### 2.3. Service robots in dentistry

Delivering intensive treatments in problematic positions, dentists may feel fatigue ultimately resulting in errors in examining oral cavity and developing subsequent and comprehensive treatment plan. Also, there is the risk of cross-contamination due to negligence or improper handling of apparatus and equipment. Digital medicine and dentistry that are suitable for robot can help to reduce this and improve oral health related quality of life comprehensively (37). These robots are used in combination with 3D mapping for invasive procedures in dentistry such as tooth preparations, autonomously placing implants, arch wire bending and teeth positioning (5, 28). Although numerous studies as presented in Table 2 show the relative application of robots in the field of dentistry but very few studies have discussed the role of UV-D robots in disinfection.

**Table 2:** Application of robots in the field of dentistry.

Author/Year	ROBOT	Application	Mechanism	Results
Yeshwante, B et al. (38)	YOMITM (Neocis, Miami, FL, USA)	First robotic computer navigation system to receive FDA approval	The navigation system uses vibrational feedback	Improves clinical precision of implant surgeries and prepares implant osteotomies with excellent predictability and precision.
Woo et al. (39)	6-DOF robotic arm	Orthognathic surgery	Movement of robotic arm in 6 different directions including translation and rotation	Provides high degree of flexibility and agility, making them suitable for tasks requiring accuracy, pace, and consistency.
Ma Q et al. (40)	OMS robot	Oral and maxillofacial surgery	A self-contained surgical system	Assists in surgery providing greater accuracy.
Zhang JT et al. (41)	Robot with novel stapler for stapling	Oral and maxillofacial surgery	Kinematics control	In addition to a novel stapler with one degree of flexibility to close the incision, this robot has six degrees of freedom for location and orientations.
Fang G et al. (42)	MR-safe soft robotic system	Head and Neck Cancer	MRI-guided	
Transoral laser microsurgery	Laser-based tumour ablation, high-contrast soft tissue imaging, comprehensive physiological change visualization, and thermometry.			
Zhang et al. (43)	6-DOF CRS robot	Teeth setting in complete denture (3D)	Scanning of the arch	Dental arch curve designing based on the patient’s jaw arch measurements and view 3D simulated teeth on a screen and adjust each tooth's position with ±0.05 mm accuracy.
Jiang, J et al. (44)	Robotic system for tooth arrangement (50 D- DOF)	Teeth-arrangement	Robotic arm in 6 different directions	Manufacturing of the full denture is finished in about 30 min with ±0.07 mm accuracy.
Alford T.J et al. (45)	SureSmile OAW bending robot	Orthodontics	Power function model.	Diagnose patients, simulating treatment plans, and personalize fixed orthodontic equipment.
Burdea et al. (46)	WidowX 250 Robot Arm 6DOF	Oral Medicine and Radiology	6-DOF robot arm, x-ray source and film	Dental subtraction radiography
Wu Y et al. (47)	Remebot Dental Robot	Prosthodontics and Oral Surgery	3D printing	Performs two distal zygomatic implants on maxilla and two immediately loaded full-arch fixed implant rehabilitation on the mandible.
Hwang G et al. in 2019 (48)	Catalytic antimicrobial robots (CARs)	Others	Dual catalytic-magnetic functionality using magnetic field powered robotic assemblies.	Create bactericidal free radicals and fighting persistent biofilm infections
P. Vela-Anton (49)	Borjibot	Pedodontics	Torsional movement	A sensitive automation system for newborn oral cognitive training that provides force and torsional stimuli
Kasimoglu Yv et al. (50)	Humanoid robots	Pedodontics	Techno-psychological distraction technique using multimodal assessment	A robot with humanoids to help Adolescents with dental fear of the dentist in children
Sakaeda et al. (51)	Shapeshifting microrobots	Public Health Dentistry	Human-centred design technology and system integration technology	An automatic teeth cleaning robot that replicated 3D brushing motions over time
Zhao R et al. (52)	Integrated dental robot system	Oral Medicine and Radiology	Robot technology	Diagnosis and maintenance care

FDA, Food and Drug Administration; MRI, magnetic resonance imaging; 3D, three dimensional; D, degree; DOF, degree of freedom.

### 2.4. Will UV-D disinfection robots complement in the field of dentistry?

Robotic dentistry has revolutionized the practice of dentistry, transforming both the mindset and approach of healthcare providers while simultaneously elevating the quality of patient care. This technological advancement has ushered in a standardized approach to harnessing robotic capabilities across various dental tasks. In the medical field, numerous studies have showcased the advantages of employing UV-D technology for disinfection purposes, extending to applications like tooth preparations and dental implant placement in prosthodontics (22), arch-wire bending robots in orthodontics, and robot-assisted craniomaxillofacial osteotomy in oral and maxillofacial surgery (25, 26). Surprisingly, despite these advancements, there have been no studies that explore the potential of UV-D robots for disinfecting dental operatories.

Despite the fact that they were developed for certain tasks such multisensory transportation and altering cells, their uses for chemical and physical biofilm destruction are still being researched (53). Hwang and colleagues (48) created catalytic antimicrobial robots (CARs) that could degrade and eliminate biofilms on surfaces in a controlled, effective, and precise manner. These “kill-grade-and-remove” CAR systems could be applied in dentistry to treat persistent biofilm infections. Thus, considering all of these changes, it becomes imperative to use a UV-D robot that is designed specifically to perform disinfection in dentistry. A new technology must overcome a number of challenges like acceptability, awareness and compliance when it is brought into a new environment. The high cost of technical breakthroughs in medical and dental applications is one such barrier. Additionally, robotic systems are sophisticated and need specialized knowledge to perform well (54). Therefore, the effectiveness of the outcomes would depend on how well the staff feed the data into the robotic system.

## 3. Discussion

### 3.1. Demonstrated success of UV-D robots in dentistry

A variety of diseases can be encountered in polluted hospital environments. Contaminated surfaces provide a risk that cannot be eradicated with the help of traditional manual methods for disinfection and cleaning (55). Furthermore, traditional disinfectants were unavailable during the COVID-19 epidemic, necessitating the use of innovative disinfectants or disinfection procedures. To solve these shortcomings, UVC disinfection machines that move autonomously, or UVC robots, have been developed (56). Dentistry is evolving into a new era of the robot-assistance based on artificial intelligence. However, these robots are still not fully incorporated into studies on dentistry. A first prototype for remote robotic dentistry was developed over the course of the past year by a group of senior biomedical engineering students at South Dakota Mines, of STEM university in South Dakota, in 2023 in collaboration with a dentist. They stated that UV-D robots could aid in improving underprivileged communities access to attain dental treatment. They also pointed out that contemporary dentistry already uses intricate and meticulous 3D scans of the oral cavity, allowing a surgery like a filling to be planned out digitally well in advance of any procedure (57). Another recently conducted research by Linn et al. in 2023 at Taipei Medical University, Taiwan employed three distinct implant sizes with 76 drilling sites in partly edentulous models, comparing the effectiveness of robotic and human unaided drilling. Algorithms for standardization and incrementally drilling techniques was used for the robotic procedure. Following the drilling process, differences between the implant’s actual position and the intended position were identified and it was further concluded that the best precision and dependability for the preoperative plan for small implant diameters can be provided by a robotic surgical system (58). A revolutionary method of interactive operative assistance, robot-assisted dental implant placement provides directions in placing implants, osteotomy preparation, and implant insertion in addition to visual navigation.

Van Riet TCT et al. in 2021 conducted a systematic review and provided a complete, transparent, and evidence-based assessment of study characteristics with state of progress of robotic initiatives in dentistry (59). Adel S et al. in 2021, conducted a scoping review and further 87 studies were added to the systematic review and stated that there has been significant research in the previous ten years on surgical robots for diagnosis, and arch wire bending. Nanorobots and rehabilitative robots hold great promise and have received a lot of attention in the orthodontic literature (60). Thus, numerous studies have been published utilizing its applications in dentistry, such as orthodontics, Prosthodontics and oral surgery, but there are very few studies suggesting its use in disinfection (59–62).

Boston Dental Clinic, a prominent dental practice in Boston, Massachusetts, and few clinics in Dubai, UAE, disinfects clinics with UV-D robots. This self-cleaning technology eliminates harmful microbes by disinfecting a 360-degree area with eight UV-C ultraviolet lasers, removing 99.99% of all viruses and bacteria. They also demonstrated that these UV-D robots are more effective than manual cleaning methods such as disinfectant spray and slow spread of COVID-19 thereby protecting frontline healthcare professionals. To protect individuals around them, the robots have a panic button and sensor-based security features that turn off UV lamps whenever a human is in close proximity to robots that are sanitizing (63). Cimolai (64) offers a comprehensive examination of COVID-19’s environmental implications and cleaning methods. In light of this, future investigations should focus on real-world assessments to detect contagious viruses. Achieving this can be simplified with UV-D robotic processors (14). These automated UV-D robots are equipped with graphical processors for object recognition and visual analysis, enhancing space sterilization efficiency. UV-D robots excel in identifying potentially contaminated objects, such as control surfaces and doorknobs, through extended exposure to UV-C light, ensuring thorough disinfection of these items.

The UVD robot’s irradiation time and speed should be adjusted to disinfect objects which are highly infested with pathogenic organisms in accordance with the results of object detection (65). Even though UV-C dosage for 99.9% COVID-19 disinfection has not been explicitly stated, it is known that under controlled laboratory circumstances, 100–200 J/m^2^ is the UV-C dose required to inactivate 99.9% of related SARS family viruses (66). First, due to the UV-C light’s (254 nm) harmful effects on nearby humans as well as possibility of bacteria regeneration or freshly contaminants at locations after sterilization, the robot’s operational duration should be monitored closely. UV-C (222 nm), a recently used wavelength for disinfection, has a lesser sterilizing efficacy but a less hazardous effect; it can be used only at certain periods when there are humans nearby (67).

The use of UV robots for disinfection raises a variety of unaddressed research problems, including better operator plans, organizing paths for maximizing UV irradiation, creating effective disinfecting procedures, and technologies to reduce UV radiation potential hazards. Thus, future hospitals’ design and inventory must provide for electronic communication between various work and patient-care systems, including those involving cleaning and disinfection robots in dental hospitals.

## 4. Conclusions

The decontamination procedure in healthcare facilities using UV-D robots is fascinating. These robots hold immense potential for future innovations, impacting sociological, public, and healthcare aspects. However, some challenges need addressing, such as improving hospital and device design for better robot visibility and movement. Additionally, customized approaches are required to determine the ideal wavelength and irradiation period for effectively inactivating specific pathogens on different surfaces. To fully integrate this innovative technology, further technical developments and clinical studies across various hospitals are essential. By leveraging the convergence of robotics and dentistry, UV-D disinfection in dental hospitals shows promising benefits and opens up a wide range of opportunities.
